# Effectiveness of Transcarotid vs Transfemoral Carotid Stenting for Stroke Prevention

**DOI:** 10.1001/jamanetworkopen.2025.9143

**Published:** 2025-04-25

**Authors:** Jesse A. Columbo, Pablo Martinez-Camblor, David H. Stone, Philip P. Goodney, Mark A. Creager, Todd A. MacKenzie, Jialin Mao, Aruna Pradhan, Sanuja Bose, Haobin Chen, A. James O’Malley, Caitlin W. Hicks

**Affiliations:** 1 Geisel School of Medicine at Dartmouth, Hanover, New Hampshire; 2 Section of Vascular Surgery, Heart and Vascular Center, Dartmouth-Hitchcock Medical Center, Lebanon, New Hampshire; 3 Department of Biomedical Data Science, Dartmouth College, Lebanon, New Hampshire; 4 Department of Anesthesia, Dartmouth Hitchcock Medical Center, Lebanon, New Hampshire; 5 Section of Cardiovascular Medicine, Heart and Vascular Center, Dartmouth Hitchcock Medical Center; 6 Dartmouth Institute for Health Policy and Clinical Practice, Dartmouth College, Lebanon, New Hampshire; 7 Department of Population Health Sciences, Weill Cornell Medical College, New York, New York; 8 Division of Preventative Medicine, Mass General Brigham, Boston, Massachusetts; 9 Division of Vascular Surgery and Endovascular Therapy, Department of Surgery, The Johns Hopkins School of Medicine, Baltimore, Maryland

## Abstract

**Importance:**

The effectiveness of surgical transcarotid artery revascularization (TCAR) compared with percutaneous transfemoral carotid artery stenting (TF-CAS) for stroke prevention beyond the periprocedural period is poorly quantified.

**Objective:**

To compare the risk of stroke after TCAR vs TF-CAS.

**Design, Setting, and Participants:**

This retrospective cohort study used data from the Vascular Implant Surveillance and Outcomes Network (VISION), a procedural registry linked to Medicare claims data that captures clinical, procedural, and outcome data on patients who underwent carotid stenting. Patients who underwent TCAR or TF-CAS between October 1, 2016, and December 31, 2019, and were captured in the VISION database were included. Data were analyzed between January and June 2024.

**Exposure:**

Type of carotid stenting (TCAR vs TF-CAS).

**Main Outcomes and Measures:**

The primary outcomes were any stroke, including both periprocedural and during follow-up, defined using a validated claims code list, and death. Asymptomatic and symptomatic patients were analyzed separately. Kaplan-Meier analysis was used to calculate the cumulative incidence of the outcomes, and a multivariable Cox proportional hazards model was used to determine hazard ratios (HRs).

**Results:**

There were 5798 asymptomatic patients (mean [SD] age, 74.6 [7.7] years; 3631 male [62.6%]; 3482 underwent TCAR; 2316 underwent TF-CAS) and 4721 symptomatic patients (mean [SD] age, 74.2 [8.3] years; 2969 male [62.9%]; 2377 underwent TCAR; 2344 underwent TF-CAS) who underwent carotid stenting. Patients who underwent TCAR were older, more likely to be female, and less likely to have had a prior ipsilateral carotid revascularization procedure. Among asymptomatic patients, the Kaplan-Meier 3-year risk of stroke was lower after TCAR (5.1%; 95% CI, 3.0%-7.1%) than TF-CAS (9.2%; 95% CI, 7.7%-10.7%) (log-rank *P* < .001). The composite 3-year stroke or death risk after TCAR was 22.6% (95% CI, 18.8%-26.3%), compared with 31.4% (95% CI, 28.3%-34.3%) after TF-CAS (log-rank *P* < .001). Compared with TCAR, the adjusted HR of stroke after TF-CAS among asymptomatic patients was 1.69 (95% CI, 1.25-2.28; *P* < .001). Among patients with symptomatic carotid stenosis, the 3-year stroke risk was also lower for TCAR (16.6%; 95% CI, 12.1%-20.9%) than for TF-CAS (20.9%; 95% CI, 17.5%-24.1%) (log-rank *P* < .001). The composite 3-year stroke or death risk after TCAR was 35.9% (95% CI, 30.1%-41.2%), compared with 41.5% (95% CI, 37.6%-45.1%) after TF-CAS (log-rank *P* < .001). Compared with TCAR, the adjusted HR for stroke after TF-CAS among symptomatic patients was 1.42 (95% CI, 1.17-1.73; *P* < .001). Sensitivity analyses yielded similar results.

**Conclusions and Relevance:**

In this comparative effectiveness study, TCAR was associated with a lower risk of stroke than TF-CAS. This finding was consistent in both asymptomatic and symptomatic patients and durable over a 3-year interval. These findings can inform procedure choices for patients considering carotid artery stenting.

## Key Points

**Question:**

What is the comparative risk of stroke after transcarotid artery revascularization (TCAR) vs transfemoral carotid artery stenting (TF-CAS) for patients with carotid artery stenosis?

**Findings:**

In this comparative effectiveness study of 5798 asymptomatic and 4721 symptomatic patients who underwent carotid stenting, TCAR was associated with a lower risk of stroke than was TF-CAS. This finding was consistent in both asymptomatic and symptomatic patients and was durable over a 3-year interval.

**Meaning:**

With no completed or enrolling randomized clinical trial to evaluate TCAR, these comparative stroke risk results can inform future procedure choice for patients who are considering carotid artery stenting.

## Teaser

This comparative effectiveness research study examines the risk of stroke among patients with carotid artery stenosis after undergoing transcarotid artery revascularization vs transfemoral carotid artery stenting.

## Introduction

In October 2023, the Centers for Medicare & Medicaid Services (CMS) expanded coverage for carotid artery stenting for beneficiaries with carotid artery stenosis and removed the strict federal oversight of quality and outcome benchmarks that had been maintained since 2005.^1^ Currently, there are 2 primary techniques for carotid stenting: percutaneous transfemoral carotid artery stenting (TF-CAS) and surgical transcarotid artery revascularization (TCAR).^2^ The efficacy and safety of TF-CAS compared with standard carotid endarterectomy (CEA) has been demonstrated in several randomized clinical trials (RCTs).^3,4,5^ By comparison, TCAR has never been part of an RCT despite the observation that it is now in use at more than 600 centers in the US.^6,7,8^

To date, observational studies comparing TCAR and TF-CAS have been limited to periprocedural outcomes, 1-year composite stroke or death rates, or mortality.^9,10,11,12^ In the absence of longer-term results, the comparative stroke risk associated with these procedures remains undefined. This knowledge gap limits both clinicians and patients in their ability to make an informed decision regarding which procedure is better for durable stroke prevention.^13^ Therefore, the purpose of this study was to define the comparative risk of stroke after TCAR vs TF-CAS for patients with carotid artery stenosis.

## Methods

### Data Source

For this comparative effectiveness research study, we used data from the Vascular Implant Surveillance and Outcomes Network (VISION). VISION is a collaborative network that combines granular clinical data on patients undergoing vascular procedures from the Vascular Quality Initiative (VQI) registry with Medicare claims data for long-term follow-up.^14^ The VQI prospectively captures demographic, clinical, procedural, and in-hospital outcomes on patients who undergo carotid stenting at participating centers.^15^ TCAR was initially approved by the US Food and Drug Administration (FDA) in 2015, and in October 2016 the VQI started the TCAR Surveillance Project (VQI-TSP) to monitor outcomes for patients undergoing this procedure.^16^ Until October 2023, CMS required that beneficiaries undergoing TCAR be entered into the VQI-TSP, tying registry entry to reimbursement; as a result, more than 95% of beneficiaries who underwent TCAR were captured.^1,9^ To create the VISION dataset used for this study, eligible patients in the VQI-TSP were linked at the individual level to their respective Medicare claims file using a validated algorithm of direct and indirect identifiers.^17^ The most recent VISION linkage included data through December 31, 2019. Therefore, we studied patients who underwent carotid artery stenting from October 1, 2016, through December 31, 2019. The Dartmouth Hitchcock Medical Center Institutional Review board approved the study. Informed consent was waived due to the retrospective study design, in accordance with 45 CFR §46. Data were analyzed from January to June 2024. The study was reported following the International Society for Pharmacoeconomics and Outcomes Research (ISPOR) reporting guidelines.^18^

### Inclusion and Exclusion Criteria

We included all patients in the VQI who underwent TCAR or TF-CAS and who were linked to their fee-for-service Medicare file. We included the first procedure performed for each patient. We excluded 174 patients who underwent stenting as part of a combined neurosurgical procedure and 188 patients who underwent stenting for an indication other than atherosclerotic or neointimal hyperplastic disease. We also excluded 221 patients who underwent stenting in the setting of an ipsilateral carotid occlusion, 13 patients missing data on whether they were asymptomatic or symptomatic at the time of their procedure, and 91 patients missing data on the procedure type.

### Exposures and Outcomes

The primary exposure was type of carotid stenting, TCAR vs TF-CAS. The primary outcome was any stroke, including ischemic and hemorrhagic events. Strokes during the hospitalization for the index procedure were identified from the VQI registry, which performs regular site audits to ensure data quality and completeness. Following discharge, strokes were detected using an externally validated list of Medicare claims codes.^19^ Secondary outcomes were death, which was obtained from the Medicare Master Beneficiary Summary File, and a composite end point of stroke or death.

### Variable Definitions

Variable definitions were determined by the VQI. Sex was defined as sex at birth. Race was self-reported and initially was defined as American Indian or Alaskan Native, Asian, Black or African American, Native Hawaiian or Other Pacific Islander, White, more than 1 race, or unknown. However, due to CMS requiring at least 11 individuals in any reported group, we combined the smaller categories so that the final categories included Asian, Black or African American, White, and unknown. Hispanic ethnicity was captured as a separate variable. Race and ethnicity were reported because prior studies^20,21^ have demonstrated differences in carotid revascularization based on these variables. Medical and anatomic high-risk conditions were defined according to the approved CMS high-risk criteria for carotid stenting.^22^ Clinical comorbid conditions were obtained according to the admission history and physical note or the preoperative clinic note. Patients were defined as taking a preoperative medication if they were reported as having taken a dose within 36 hours of their procedure.

### Statistical Analysis

We examined demographic, clinical, and procedural characteristics of patients undergoing TCAR and TF-CAS. We reported means with SDs for continuous variables and compared them with *t* tests. We reported counts with percentages for categorical variables and compared them with χ^2^ tests. We used Kaplan-Meier analysis to calculate the cumulative incidence of the outcomes. We censored patients at the end of the study period, end of fee-for-service coverage, or death (for stroke model only), whichever was earliest. We used a multivariable Cox proportional hazards model in our primary analysis to compare outcomes between patients undergoing TCAR and TF-CAS and presented hazard ratios (HRs) and 95% CIs. TCAR served as the reference category for all estimates. The following variables were chosen a priori for inclusion in the model on the basis of previous studies^7,8,9,10,23,24^: age, sex, race, Hispanic ethnicity, coronary artery disease, congestive heart failure, prior coronary revascularization, hypertension, chronic obstructive pulmonary disease, diabetes, dialysis, smoking status, prior ipsilateral or contralateral carotid procedure, anatomic or medical high risk, severity of carotid stenosis, preoperative medications (including aspirin, P2Y_12_ inhibitors, dual antiplatelet therapy, statins, β-blockers, angiotensin-converting enzyme inhibitors, and anticoagulants), procedure year, annual proceduralist-level and center-level carotid stent volumes, and the treating center as a random effect. We analyzed outcomes for asymptomatic and symptomatic patients separately.

We conducted 4 sensitivity analyses to test whether our findings were sensitive to a specific statistical modeling technique. First, we created propensity score–matched groups of patients who underwent TCAR or TF-CAS using the same covariates as in the Cox model, for asymptomatic and symptomatic patients, respectively. This allowed us to compare the risk of stroke for TCAR vs TFCAS among patients who were similar in observed characteristics, relying less on the Cox model for risk adjustment. Second, because unmeasured confounding remained a concern, we used a 2-stage residual inclusion instrumental variable analysis.^25,26^ Our instrument was the proportion of TCAR out of the total carotid stenting procedures performed at each center over the 365 days before the admission date of each patient. The instrumental variable analysis relies on several assumptions that we believe are met in this setting, as described elsewhere.^10,27^ Third, because patients who underwent TCAR had a lower rate of mortality than those who underwent TF-CAS, we used a Fine-Gray competing risk estimator to determine the subdistribution HR for stroke with death as a competing risk.^28^ Fourth, because there was evidence of nonproportional hazards for stroke, with most strokes occurring early during follow-up, we calculated time-stratified HRs for stroke: one from the procedure date to 30 days, and one from 31 days through 3 years.^29,30^ R statistical software version 4.4.3 (R Project for Statistical Computing) was used for all analyses. All *P* values were 2 tailed, and *P* < .05 denoted statistical significance.

## Results

### Asymptomatic Patients

#### Baseline Cohort Characteristics

Of a total of 5798 asymptomatic patients (mean [SD] age, 74.6 [7.7] years; 3631 male [62.6%]), 3482 underwent TCAR (mean [SD] age, 75.5 [7.1] years; 1329 female [38.2%]) and 2316 underwent TF-CAS (mean [SD] age, 73.2 [7.6] years; 838 female [36.2%]) (Table 1 and eFigure in Supplement 1). Patients who underwent TCAR were more likely to meet anatomic high-risk criteria (TCAR vs TF-CAS, 2065 patients [59.8%] vs 1023 patients [44.5%]), but were less likely to have undergone a prior ipsilateral carotid procedure (TCAR vs TF-CAS, 533 patients [15.3%] vs 569 patients [24.6%]).

**Table 1.** Cohort Characteristics

Characteristic	Asymptomatic patients (n = 5798)			Symptomatic patients (n = 4271)		
	TF-CAS (n = 2316), No. (%)	TCAR (n = 3482), No. (%)	*P* value	TF-CAS (n = 2344), No. (%)	TCAR (n = 2377), No. (%)	*P* value
Demographics						
Age, mean (SD), y	73.2 (7.6)	75.5 (7.2)	<.001	73.0 (8.2)	75.4 (7.9)	<.001
Sex						
Female	838 (36.2)	1329 (38.2)	.13	851 (36.3)	901 (37.9)	.27
Male	1478 (63.8)	2153 (61.8)		1493 (63.7)	1476 (62.1)	
Race						
Asian	26 (1.1)	20 (0.6)	.03	16 (0.7)	17 (0.7)	>.99
Black or African American	97 (4.2)	97 (2.8)	.005	142 (6.1)	90 (3.8)	<.001
White	2129 (92.0)	3253 (93.5)	.03	2113 (90.1)	2204 (92.8)	.001
Unknown^a^	63 (2.7)	110 (3.2)	.38	73 (3.1)	63 (2.7)	.39
Hispanic ethnicity	44 (1.9)	93 (2.7)	.07	68 (2.9)	59 (2.5)	.44
Clinical characteristics						
Coronary artery disease	1468 (64.6)	1935 (55.7)	<.001	1038 (44.3)	1162 (48.9)	.002
Congestive heart failure	433 (18.7)	626 (18.0)	.51	447 (19.1)	435 (18.3)	.52
Moderate or severe	103 (4.5)	116 (3.3)	.04	89 (3.8)	71 (3.0)	.15
Prior coronary revascularization	1055 (45.7)	1537 (44.2)	.30	856 (36.5)	903 (38.0)	.31
Hypertension	2050 (90.6)	3147 (90.5)	.93	2105 (89.8)	2167 (91.3)	.10
Chronic obstructive pulmonary disease	680 (29.4)	942 (27.1)	.06	683 (29.1)	675 (28.4)	.60
Receiving home oxygen	121 (5.2)	141 (4.1)	.04	109 (4.7)	100 (4.2)	.51
Diabetes	920 (40.0)	1228 (35.3)	<.001	906 (38.7)	939 (39.5)	.57
Taking insulin	350 (15.2)	413 (11.9)	<.001	379 (16.2)	379 (16.0)	.87
Dialysis	38 (1.6)	51 (1.5)	.67	44 (1.9)	57 (2.4)	.25
Smoking						
Never	602 (26.0)	937 (26.9)	.46	650 (27.7)	658 (27.7)	>.99
Prior	1206 (52.1)	1903 (54.7)	.06	1104 (47.1)	1251 (52.7)	<.001
Current	507 (21.9)	640 (18.4)	.001	589 (25.1)	465 (19.6)	<.001
Prior ipsilateral carotid procedure	569 (24.6)	533 (15.3)	<.001	478 (20.4)	335 (14.1)	<.001
Prior contralateral carotid procedure	396 (17.1)	628 (18.1)	.38	390 (16.7)	422 (17.8)	.34
Anatomic high risk^b^	1023 (44.5)	2065 (59.8)	<.001	1052 (45.4)	1436 (60.8)	<.001
Medical high risk^b^	943 (41.1)	1517 (43.9)	.03	1004 (43.3)	1050 (44.5)	.43
Both	335 (14.6)	688 (19.9)	<.001	341 (14.7)	465 (19.7)	<.001
Degree of carotid stenosis, %						
0-69	157 (6.8)	192 (5.5)	.05	261 (11.1)	300 (12.6)	.13
≥70	1556 (67.2)	3121 (89.6)	<.001	2010 (85.8)	2001 (84.2)	.14
Unknown	603 (26.0)	169 (4.9)	<.001	73 (3.1)	76 (3.2)	.94
Preoperative medications						
Aspirin	2012 (86.9)	3120 (89.6)	.002	2040 (87.0)	2141 (90.1)	.001
P2Y_12_ inhibitor	1811 (78.3)	2964 (85.2)	<.001	1792 (76.5)	2090 (87.9)	<.001
Dual antiplatelet	1632 (70.5)	2713 (78.0)	<.001	1642 (70.1)	1929 (81.2)	<.001
Statin	1883 (81.4)	3091 (88.8)	<.001	1934 (82.5)	2139 (90.0)	<.001
β-blocker	1382 (59.7)	2056 (59.0)	.66	1257 (53.6)	1359 (57.2)	.02
Anticoagulation	317 (13.7)	480 (13.8)	.95	367 (15.7)	393 (16.5)	.44
Angiotensin-converting enzyme inhibitor	1140 (49.3)	1849 (53.2)	.004	1110 (47.4)	1181 (49.7)	.12
Stenting volumes						
Procedure year						
2016	266 (11.5)	44 (1.3)	<.001	53 (2.3)	12 (0.5)	<.001
2017	827 (35.7)	465 (13.4)	<.001	650 (27.7)	287 (12.1)	<.001
2018	570 (24.6)	1049 (30.1)	<.001	829 (35.4)	748 (31.5)	.005
2019	653 (28.2)	1924 (55.3)	<.001	812 (34.6)	1330 (56.0)	<.001
Annual proceduralist carotid stent volumes						
Quartile 1	297 (12.8)	219 (6.3)	<.001	314 (13.4)	160 (6.7)	<.001
Quartile 2	334 (14.4)	255 (7.3)	<.001	323 (13.8)	212 (8.9)	<.001
Quartile 3	466 (20.1)	524 (15.0)	<.001	448 (19.1)	367 (15.4)	.001
Quartile 4	1219 (52.6)	2484 (71.3)	<.001	1259 (53.7)	1638 (68.9)	<.001
Annual center carotid stent volume						
Quartile 1	219 (9.5)	161 (4.6)	<.001	138 (5.9)	100 (4.2)	.01
Quartile 2	257 (11.1)	277 (8.0)	<.001	210 (9.0)	189 (8.0)	.23
Quartile 3	578 (25.0)	826 (23.7)	.30	538 (23.0)	588 (24.7)	.16
Quartile 4	1262 (54.5)	2218 (63.7)	<.001	1458 (62.2)	1500 (63.1)	.54

Abbreviations: TCAR, transcarotid artery stenting; TF-CAS, transfemoral carotid artery stenting.

^a^ This category includes American Indian or Alaskan Native, Native Hawaiian or Other Pacific Islander, more than 1 race, and unknown. They were combined because of Centers for Medicare & Medicaid Services rules requiring at least 11 individuals in any reported group.

^b^ Meets Centers for Medicare & Medicaid Services high-risk criteria.

#### Stroke and Death After TCAR and TF-CAS

**Figure 1.**
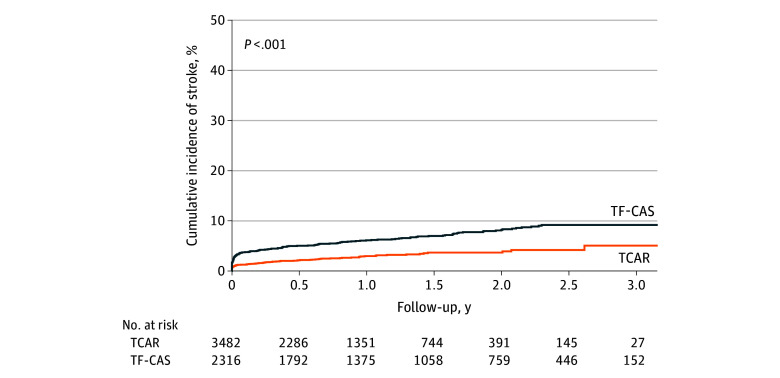
Cumulative Incidence of Stroke Among Asymptomatic Patients Undergoing Transcarotid Artery Revascularization (TCAR) or Transfemoral Carotid Artery Stenting (TF-CAS)

The 3-year Kaplan-Meier stroke risk among asymptomatic patients was 5.1% (95% CI, 3.0%-7.1%) after TCAR and 9.2% (95% CI, 7.7%-10.7%) after TF-CAS (log-rank *P* < .001) (Figure 1 and Table 2). Compared with TCAR, the adjusted HR of stroke after TF-CAS was 1.69 (95% CI, 1.25-2.28; *P* < .001) (Table 3). The adjusted HR of stroke in the first 30 days after the procedure was 2.09 (95% CI, 1.36-3.22; *P* < .001) and that from 31 days to 3 years was 1.40 (95% CI, 0.91-2.14; *P* = .12). The sensitivity analyses using propensity scores, instrumental variable analyses, and competing-risk methods were consistent with the primary results (Table 3), and the propensity score–matched groups were well balanced (eTable 1 in Supplement 1).

**Table 2.** Cumulative Incidence of Stroke and Death After TF-CAS and TCAR

Group, outcome, and time	Cumulative incidence, % (95% CI)	
	TCAR	TF-CAS
Asymptomatic patients		
Stroke		
1 y	3.4 (2.3-2.6)	6.1 (5.1-7.2)
2 y	3.7 (2.8-4.5)	8.2 (6.9-9.5)
3 y	5.1 (3.0-7.1)	9.2 (7.7-10.7)
Stroke or death		
1 y	8.6 (7.5-9.7)	13.6 (12.1-15.1)
2 y	16.1 (13.9-18.1)	23.0 (20.9-25.0)
3 y	22.6 (18.8-26.3)	31.4 (28.3-34.3)
Death		
1 y	6.4 (5.4-7.4)	8.9 (7.7-10.2)
2 y	13.8 (11.7-15.8)	18.1 (16.1-20.0)
3 y	21.5 (16.5-26.1)	26.2 (23.3-29.0)
Symptomatic patients		
Stroke		
1 y	9.8 (8.4-11.1)	12.1 (10.7-13.5)
2 y	11.7 (9.9-13.5)	16.8 (14.7-18.7)
3 y	16.6 (12.1-20.9)	20.9 (175-24.1)
Stroke or death		
1 y	16.9 (15.1-18.7)	21.7 (19.9-23.5)
2 y	26.0 (23.2-28.8)	33.5 (30.8-36.0)
3 y	35.9 (30.1-41.2)	41.5 (37.6-45.1)
Death		
1 y	10.7 (9.2-12.3)	13.2 (11.7-14.7)
2 y	19.3 (16.5-21.9)	23.0 (20.7-25.3)
3 y	25.4 (20.4-30.1)	30.9 (27.2-34.4)

Abbreviations: TCAR, transcarotid artery revascularization; TF-CAS, transfemoral carotid artery stenting.

**Table 3.** HRs of Stroke for Transfemoral Carotid Artery Stenting vs Transcarotid Artery Revascularization (Reference Value) Among Asymptomatic and Symptomatic Patients

Variable	Stroke	
	HR (95% CI)	*P* value
Asymptomatic patients		
Unadjusted HR	2.19 (1.70-2.83)	<.001
Cox-adjusted HR	1.69 (1.25-2.28)	<.001
Propensity score–matched HR	1.71 (1.20-2.46)	<.001
Instrumental variable–adjusted HR	2.41 (1.54-3.75)	<.001
Subdistribution HR (Fine-Gray)	1.68 (1.22-2.32)	<.001
Symptomatic patients		
Unadjusted HR	1.34 (1.12-1.61)	<.001
Cox-adjusted HR	1.42 (1.17-1.73)	<.001
Propensity score–matched HR	1.53 (1.22-1.93)	<.001
Instrumental variable–adjusted HR	1.63 (1.24-2.14)	<.001
Subdistribution HR (Fine-Gray)	1.41 (1.17-1.69)	<.001

Abbreviation: HR, hazard ratio.

The 3-year mortality was 21.5% (95% CI, 16.5%-26.1%) after TCAR and 26.2% (95% CI, 23.3%-29.0%) after TF-CAS (log-rank *P* < .001). The 3-year risk of stroke or death as a composite outcome was 22.6% (95% CI, 18.8%-26.3%) after TCAR and 31.4% (95% CI, 28.3%-34.3%) after TF-CAS (log-rank *P* < .001).

### Symptomatic Patients

#### Baseline Cohort Characteristics 

Of a total of 4721 symptomatic patients (mean [SD] age, 74.2 [8.3] years; 2969 male [62.9%]), 2377 underwent TCAR and 2344 underwent TF-CAS. Symptomatic patients who underwent TCAR were older than those who underwent TF-CAS (mean [SD] age, 75.4 [7.9] years vs 73.0 [8.2] years), were more likely to be female (TCAR vs TF-CAS, 901 female patients [37.9%] vs 851 female patients [36.3%]), and were more likely to meet anatomic high-risk criteria (TCAR vs TF-CAS, 1436 patients [60.8%] vs 1050 patients [45.5%]), but were less likely to have undergone a prior ipsilateral carotid procedure (TCAR vs TF-CAS, 335 patients [14.1%] vs 478 patients [20.4%]) (Table 1).

#### Stroke and Death After TCAR and TF-CAS

**Figure 2.**
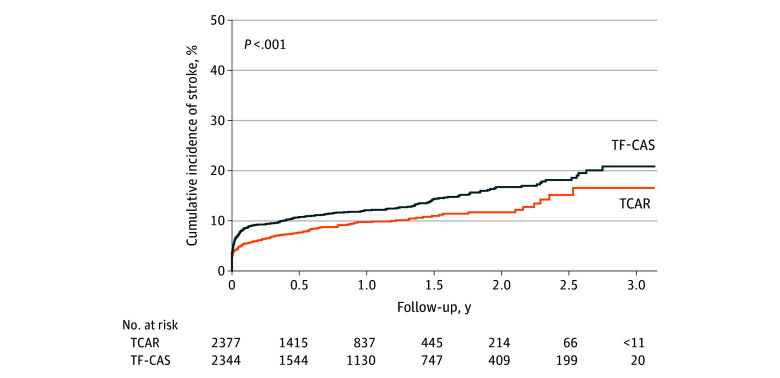
Cumulative Incidence of Stroke Among Symptomatic Patients Undergoing Transcarotid Artery Revascularization (TCAR) or Transfemoral Carotid Artery Stenting (TF-CAS)

The 3-year Kaplan-Meier stroke risk among symptomatic patients was 16.6% (95% CI, 12.1%-20.9%) after TCAR and 20.9% (95% CI, 17.5%-24.1%) after TF-CAS (log-rank *P* < .001) (Figure 2). Compared with TCAR, the adjusted HR of stroke after TF-CAS was 1.42 (95% CI, 1.17-1.73; *P* < .001). The adjusted HR of stroke in the first 30 days after the procedure was 1.57 (95% CI, 1.22-2.03; *P* < .001) and that from 31 days to 3 years was 1.24 (95% CI, 0.92-1.68; *P* = .17). The sensitivity analyses using propensity scores, instrumental variable analyses, and competing risk methods were consistent with the primary results (Table 3), and the propensity score–matched groups were well balanced (eTable 2 in Supplement 1).

The 3-year mortality was 25.4% (95% CI, 20.4%-30.1%) after TCAR and 30.9% (95% CI, 27.2%-34.4%) after TF-CAS (log-rank *P* < .001). The 3-year rate of stroke or death as a composite outcome was 35.9% (95% CI, 30.1%-41.2%) after TCAR and 41.5% (95% CI, 37.6%-45.1%) after TF-CAS (log-rank *P* < .001).

## Discussion

To our knowledge, this comparative effectiveness research study is the first to report the 3-year risk of stroke for TCAR vs TF-CAS using the VISION observational registry and a validated set of claims codes for stroke detection. We determined that the risk of stroke was lower among patients who underwent TCAR compared with those who underwent TF-CAS. This finding was largely associated with differences in perioperative stroke outcomes, but was durable over the 3 years of documented follow-up and was consistent among both asymptomatic and symptomatic patients. These findings can inform future procedure choices for patients considering carotid artery stenting.

Our study findings are of particular importance to inform contemporary practice in the context of the rapidly evolving clinical treatment paradigms for carotid artery stenosis in the US. In addition to optimal medical therapy and risk factor modification, carotid revascularization with CEA is supported by several RCTs for stroke-risk reduction in appropriate patients.^2,31,32,33,34,35,36,37,38^ In the 1990s, TF-CAS emerged as an alternative for patients considered high-risk for CEA. CMS reimbursement for TF-CAS was predicated on strict operator-based competency with associated quality benchmarks, and participation in a formal clinical trial or registry was mandatory.^39^ Several RCTs were subsequently conducted comparing TF-CAS with CEA and demonstrated acceptably low stroke rates in those defined settings.^3,4^

In 2015, the FDA approved TCAR as an alternative method of carotid stenting, expanding the therapeutic options for high-risk patients.^40^ No RCT was thought to be necessary owing to TCAR’s inherent similarities to TF-CAS, and, accordingly, both procedures were subject to the same reimbursement restrictions.^40,41^ Early observational results for TCAR, which included both symptomatic and asymptomatic patients, documented a periprocedural stroke risk of 1.9%, with a relative risk compared with TF-CAS of 0.51 (95% CI, 0.37-0.72).^9,42^ This low stroke risk was consistent for both experienced and new proceduralists alike, a notable difference from TF-CAS.^39,42,43^ On the basis of these observational findings, the FDA expanded its approval for TCAR to include standard-risk patients in 2022.^44^ In October 2023, CMS removed federal regulatory stipulations surrounding both TCAR and TF-CAS and assigned oversight responsibility to individual institutions.^1^

With no RCT to document the efficacy of TCAR, and receding regulatory oversight for carotid artery stenting, high-quality observational studies are required to inform the optimal roles for each procedure in current practice. Prior large studies of TCAR have only reported perioperative stroke results, a composite of stroke or death at 1 year, or focused solely on mortality.^9,10,12^ Our study now adds 3-year data using VISION to document the risk of stroke after TCAR vs TF-CAS and, to our knowledge, represents the longest available stroke follow-up comparing the 2 procedures in a large cohort of patients. Our results demonstrate that TCAR is associated with a decreased risk of stroke compared with TF-CAS, confirming prior periprocedural TCAR outcome studies and documenting comparatively low stroke risk over a 3-year period.^9,10^ These results support a growing role for TCAR in evidence-based treatment paradigms.

There are 2 primary mechanisms that may explain the observed decreased stroke risk associated with TCAR compared with TF-CAS. The divergence in the risk of stroke between the 2 procedures primarily appeared in the periprocedural period, which suggests that there are significant risk differences in the conduct of the 2 stenting procedures, rather than longer term. TCAR is performed via a low-neck incision with direct carotid artery access, obviating the need to traverse the aortic arch that is required with TF-CAS, which may be a risk factor for embolic stroke.^45,46^ In addition, TCAR is performed using flow-reversal for embolic protection, which can be initiated before manipulation of the carotid lesion.^45^ In contrast, TF-CAS is primarily performed using distal filter embolic protection, during which the carotid lesion must be first crossed before filter deployment, and this maneuver may be associated with stroke.^47,48,49^ These technical differences may account for the observed reduction in stroke risk between TCAR and TF-CAS, which predominantly occurred in the perioperative period.

The risk of stroke and mortality documented in the current study is higher than that reported by many trials of TF-CAS.^3,4,50^ RCTs are highly supervised, and carotid stent proceduralists must undergo rigorous evaluation and meet strict operator competency and volume benchmarks, often with a lead-in of proctored procedures, before enrolling patients in the trial.^3,4,51^ Results from these RCTs often represent the best case for possible results with a given procedure and may not be generalizable to everyday clinical practice or populations not tested in the trial.^52^ Realizing this, CMS had implemented strict operator-based competency benchmarks in 2005 to closely monitor outcomes of carotid stenting for Medicare beneficiaries, although these are not as strict as those required in major RCTs.^3,4,39,51^ Results from clinical practice studies, such as this one, can offer a complementary perspective to the results of RCTs, documenting how the procedures may perform once they are in widespread use.^52,53^ The higher stroke and mortality risks we report provide the sobering view that carotid stenting procedures may not perform as well in clinical practice as may be anticipated on the basis of the results of RCTs. Nevertheless, our results are consistent with prior studies of TF-CAS and CEA in the Medicare population, supporting the validity of the clinical practice risks documented here.^54,55^ It is, therefore, imperative that carotid revascularization be applied judiciously and after careful consideration of the risks and benefits with each patient.^13,56^

### Limitations

This study has several limitations. It is subject to the inherent limitations of observational research methods. To address these, we conducted several sensitivity analyses, each with different underlying assumptions. The point estimates from all of the modeling techniques were in the same direction, which we believe strengthens our conclusion that the risk of stroke is lower for TCAR than for TF-CAS. The proportional use of TCAR increased over the study period. We performed a sensitivity analysis evaluating the time-stratified HR for stroke broken into the first 30 days after the procedure and from 31 days to 3 years after the procedure since more patients undergoing TCAR were likely censored than those undergoing TF-CAS. This sensitivity analysis demonstrated that the significant differences in outcomes are largely associated with the perioperative differences, rather than the long-term outcomes. Participation in the VQI is voluntary, and outcomes such as stroke are self-reported by treating physicians, which may bias the periprocedural documented stroke risk. However, we anticipate that this would bias both TCAR and TF-CAS similarly. Furthermore, this should not impact the detection of stroke after discharge, which relied on validated Medicare claims capture. Our study only included patients who were linked to Medicare, and results may not be generalizable to non-Medicare patients or non-VQI centers. We relied on the use of claims codes to identify stroke events after the periprocedural period. This approach has been validated in 2 external cohorts with expert-adjudicated stroke events, but event capture may be overestimated particularly among symptomatic patients.^19^ Nevertheless, to our knowledge, this is the first study to use VISION data to study the comparative effectiveness of stroke risk after TCAR vs TF-CAS in current practice.

## Conclusions

In this comparative effectiveness study using the VISION registry linked to Medicare claims for follow-up, we determined that the risk of stroke was lower among patients who underwent TCAR compared with those who underwent TF-CAS. This finding was durable over 3 years of follow-up, and among both asymptomatic and symptomatic patients. In the absence of an RCT, these data represent important findings to inform future procedure choices for patients considering carotid stenting and add to the growing body of literature to support the growing role of TCAR in the treatment of carotid artery stenosis.

## Notes

Supplement 1. **eFigure**. Flow diagram of cohort creation **eTable 1.** Cohort characteristics of asymptomatic propensity score matched cohort **eTable 2.** Cohort characteristics of symptomatic propensity score matched cohort

Supplement 2.
Data Sharing Statement
