# Supplementary material for: Effectiveness of Transcarotid vs Transfemoral Carotid Stenting for Stroke Prevention
Source: JAMA Netw Open. 2025 Apr 25;8(4):e259143. doi: 10.1001/jamanetworkopen.2025.9143 (PMC12032571; doi:10.1001/jamanetworkopen.2025.9143)
Supplement: Supplement 1. — eFigure. Flow diagram of cohort creation eTable 1. Cohort characteristics of asymptomatic propensity score matched cohort eTable 2. Cohort characteristics of symptomatic propensity score matched cohort [file jamanetwopen-e259143-s001.pdf]

Supplemental Online Content

Columbo JA, Martinez-Camblor P, Stone DH, et al. Effectiveness of transcarotid vs transfemoral carotid stenting for stroke prevention. *JAMA Netw Open*. 2025;8(4):e259143. doi:10.1001/jamanetworkopen.2025.9143

eFigure. Flow diagram of cohort creation
eTable 1. Cohort characteristics of asymptomatic propensity score matched cohort
eTable 2. Cohort characteristics of symptomatic propensity score matched cohort

This supplemental material has been provided by the authors to give readers additional information about their work.

© 2025 Columbo JA et al. *JAMA Network Open*.

eFigure. Flow diagram of cohort creation

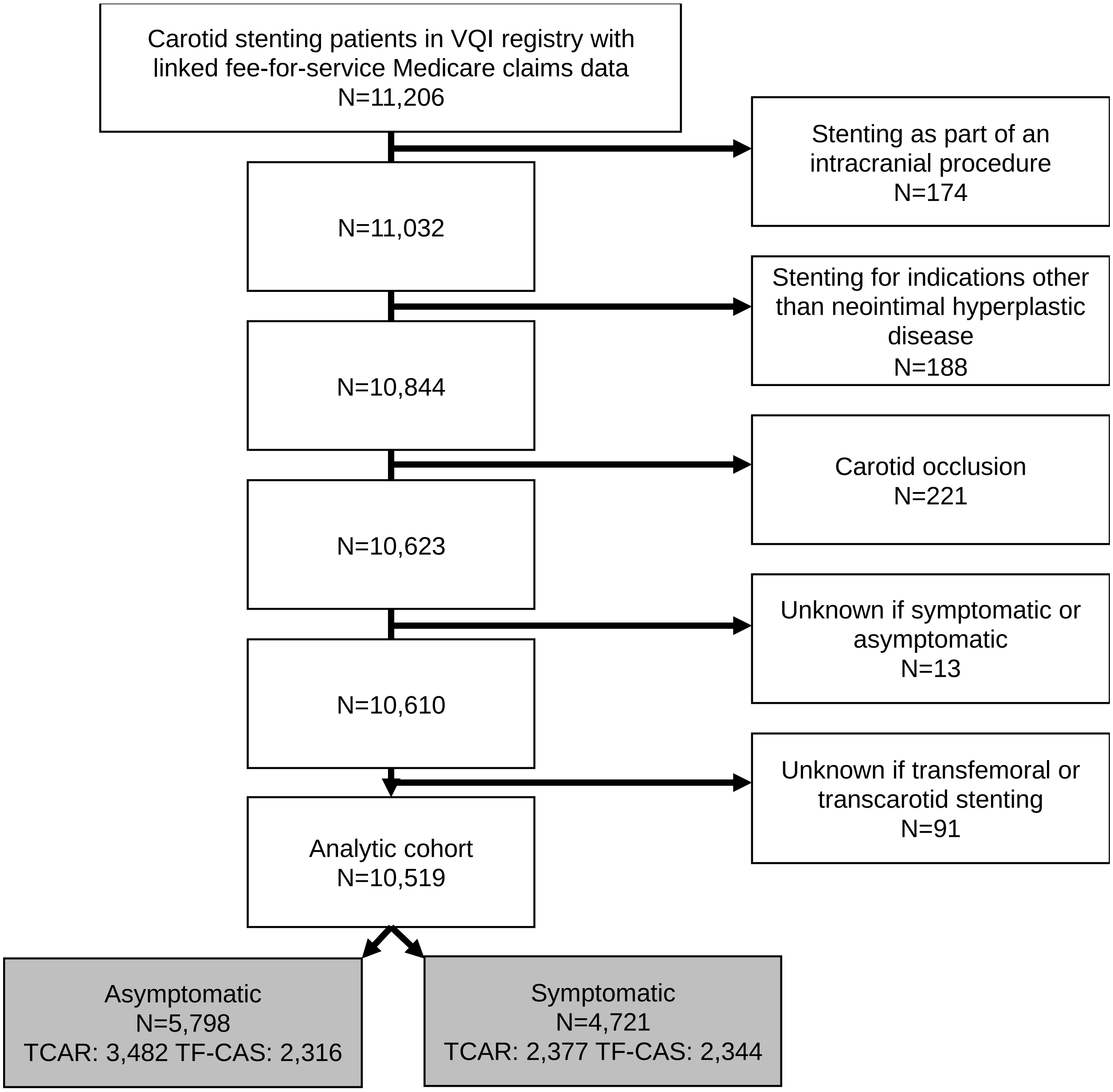

Legend: VQI, vascular quality initiative; TCAR, transcarotid artery revascularization; TF-CAS, transfemoral carotid artery stenting.

© 2025 Columbo JA et al. *JAMA Network Open*.

eTable 1: Cohort characteristics of asymptomatic propensity score matched cohort

	PS-Matched			
Demographics	TF-CAS N (%) n=1569	TCAR N (%) n=1560	p-Value	SMD
Age, mean±sd (years)	73.9±7.4	74.0±7.2	0.960	-0.02
Female	548 (35.7)	560 (36.5)	0.582	-0.02
Race				
Asian	18 (1.2)	<11 (<1)	0.679	0.06
Black or African American	54 (3.5)	50 (3.3)	0.765	0.01
Unknown	35 (2.3)	35 (2.3)	0.888	0.00
White	1424 (92.8)	1427 (93.0)	0.122	-0.01
Hispanic	39 (2.5)	49 (3.2)	1.000	-0.04
Clinical Characteristics				
Coronary artery disease	880 (57.3)	887 (57.8)	0.330	-0.01
Congestive heart failure	292 (19.0)	293 (19.1)	0.827	0.00
Moderate/severe	67 (4.4)	74 (4.8)	1.000	-0.02
Prior coronary revascularization	689 (44.9)	692 (45.1)	0.605	0.00
Hypertension	1396 (90.9)	1390 (90.6)	0.942	0.01
COPD	456 (29.7)	453 (29.5)	0.755	0.00
On home oxygen	76 (5.0)	77 (5.0)	0.937	0.00
Diabetes	595 (38.8)	621 (40.5)	1.000	-0.03
On insulin	214 (13.9)	240 (15.6)	0.356	-0.05
Dialysis	25 (1.6)	23 (1.5)	0.204	0.01
Smoking				
Never	393 (25.6)	395 (25.7)	0.884	0.00
Prior	811 (52.8)	813 (53.0)	0.967	0.00
Current	331 (21.6)	327 (21.3)	0.971	0.01
Prior ipsilateral carotid procedure	347 (22.6)	336 (21.9)	0.895	0.02
Prior contralateral carotid procedure	288 (18.8)	293 (19.1)	0.664	-0.01
Anatomic high risk[a]	779 (50.7)	779 (50.7)	0.854	0.00
Medical high risk[a]	653 (42.5)	660 (43.0)	1.000	-0.01
Both	276 (18.0)	238 (15.5)	0.827	0.07
Degree of carotid stenosis				
0-69%	117 (7.6)	103 (6.7)	0.074	0.04
≥70%	1297 (84.5)	1296 (84.4)	0.363	0.00
Unknown	121 (7.9)	136 (8.9)	1.000	-0.04
Preoperative medications				
Aspirin	1351 (88.0)	1357 (88.4)	0.362	-0.01
P2Y12 inhibitor	1232 (80.3)	1243 (81.0)	0.780	-0.02
Dual antiplatelet	1110 (72.3)	1129 (73.6)	0.648	-0.03
Statin	1293 (84.2)	1294 (84.3)	0.465	0.00
Beta-blocker	915 (59.6)	912 (59.4)	1.000	0.00
Anticoagulation	216 (14.1)	211 (13.7)	0.941	0.01
ACE inhibitor	773 (50.4)	760 (49.5)	0.835	0.02
Stenting Volumes				
Procedure year				
2016	33 (2.1)	40 (2.6)	0.477	-0.03
2017	385 (25.1)	367 (23.9)	0.476	0.03
2018	503 (32.8)	514 (33.5)	0.701	-0.02
2019	614 (40.0)	614 (40.0)	1.000	0.00
Annual proceduralist carotid stent volumes				

© 2025 Columbo JA et al. *JAMA Network Open*.

Quartile 1	144 (9.4)	144 (9.4)	1.000	0.00
Quartile 2	173 (11.3)	175 (11.4)	0.955	0.00
Quartile 3	305 (19.9)	304 (19.8)	1.000	0.00
Quartile 4	913 (59.5)	912 (59.4)	1.000	0.00
Annual center carotid stent volume				
Quartile 1	96 (6.3)	87 (5.7)	0.542	0.02
Quartile 2	126 (8.2)	139 (9.1)	0.441	-0.03
Quartile 3	385 (25.1)	394 (25.7)	0.740	-0.01
Quartile 4	928 (60.5)	915 (59.6)	0.658	0.02

[a]Meets Centers for Medicare and Medicaid Services high risk criteria.
Legend: PS, propensity score; TF-CAS, transfemoral carotid artery stenting; TCAR, transcarotid artery stenting; SMD, standardized mean difference; SD, standard deviation; COPD, chronic obstructive pulmonary disease.

© 2025 Columbo JA et al. *JAMA Network Open*.

eTable 2: Cohort characteristics of symptomatic propensity score matched cohort

	PS-Matched			
Demographics	**TF-CAS N (%) n=1569**	**TCAR N (%) n=1560**	**p-Value**	**SMD**
Age, mean±sd (years)	74.3±8.0	74.3±8.0	0.980	0.00
Female	560 (36.6)	537 (35.1)	0.407	0.03
Race				
Asian	<11 (<1)	<11 (<1)	1.000	-0.01
Black or African American	75 (4.9)	74 (4.8)	1.000	0.00
Unknown	>39 (3.5)	>39 (2.5)	1.000	0.00
White	1405 (91.7)	1404 (91.6)	1.000	0.00
Hispanic	43 (2.8)	44 (2.9)	1.000	0.00
Clinical Characteristics				
Coronary artery disease	717 (46.8)	718 (46.9)	1.000	0.00
Congestive heart failure	292 (19.1)	299 (19.5)	0.784	-0.01
Moderate/severe	58 (3.8)	57 (3.7)	1.000	0.00
Prior coronary revascularization	572 (37.3)	575 (37.5)	0.940	0.00
Hypertension	1391 (90.8)	1387 (90.5)	0.852	0.01
COPD	451 (29.4)	455 (29.7)	0.905	-0.01
On home oxygen	71 (4.6)	68 (4.4)	0.862	0.01
Diabetes	606 (39.6)	585 (38.2)	0.459	0.03
On insulin	260 (17.0)	244 (15.9)	0.465	0.03
Dialysis	34 (2.2)	29 (1.9)	0.611	0.02
Smoking				
Never	427 (27.9)	429 (28.0)	0.968	0.00
Prior	765 (49.9)	743 (48.5)	0.448	0.03
Current	340 (22.2)	360 (23.5)	0.414	-0.03
Prior ipsilateral carotid procedure	267 (17.4)	280 (18.3)	0.571	-0.02
Prior contralateral carotid procedure	262 (17.1)	261 (17.0)	1.000	0.00
Anatomic high risk[a]	814 (53.1)	821 (53.6)	0.828	-0.01
Medical high risk[a]	671 (43.8)	696 (45.4)	0.383	-0.03
Both	274 (17.9)	265 (17.3)	0.704	0.02
Degree of carotid stenosis				
0-69%	182 (11.9)	185 (12.1)	0.911	-0.01
≥70%	1310 (85.5)	1302 (85.0)	0.721	0.01
Unknown	40 (2.6)	45 (2.9)	0.660	-0.02
Preoperative medications				
Aspirin	1370 (89.4)	1360 (88.8)	0.602	0.02
P2Y12 inhibitor	1288 (84.1)	1290 (84.2)	0.961	0.00
Dual antiplatelet	1183 (77.2)	1179 (77.0)	0.897	0.01
Statin	1345 (87.8)	1342 (87.6)	0.912	0.01
Beta-blocker	847 (55.3)	860 (56.1)	0.663	-0.02
Anticoagulation	237 (15.5)	234 (15.3)	0.920	0.01
ACE inhibitor	746 (48.7)	745 (48.6)	1.000	0.00
Stenting Volumes				
Procedure year				
2016	13 (0.8)	12 (0.8)	1.000	0.01
2017	277 (18.1)	267 (17.4)	0.670	0.02
2018	545 (35.6)	569 (37.1)	0.388	-0.03
2019	697 (45.5)	684 (44.6)	0.663	0.02
Annual proceduralist carotid stent volumes				

© 2025 Columbo JA et al. *JAMA Network Open*.

Quartile 1	142 (9.3)	134 (8.7)	0.659	0.02
Quartile 2	175 (11.4)	176 (11.5)	1.000	0.00
Quartile 3	276 (18.0)	275 (18.0)	1.000	0.00
Quartile 4	939 (61.3)	947 (61.8)	0.795	-0.01
Annual center carotid stent volume				
Quartile 1	72 (4.7)	70 (4.6)	0.932	0.01
Quartile 2	121 (7.9)	128 (8.4)	0.692	-0.02
Quartile 3	377 (24.6)	359 (23.4)	0.472	0.03
Quartile 4	962 (62.8)	975 (63.6)	0.653	-0.02

[a]Meets Centers for Medicare and Medicaid Services high risk criteria.
Legend: PS, propensity score; TF-CAS, transfemoral carotid artery stenting; TCAR, transcarotid artery stenting; SMD, standardized mean difference; SD, standard deviation; COPD, chronic obstructive pulmonary disease.

© 2025 Columbo JA et al. *JAMA Network Open*.
