# Supplementary material for: Effectiveness of Transcarotid vs Transfemoral Carotid Stenting for Stroke Prevention
Source: JAMA Netw Open. 2025 Apr 25;8(4):e259143. doi: 10.1001/jamanetworkopen.2025.9143 (PMC12032571; doi:10.1001/jamanetworkopen.2025.9143)
Supplement: Supplement 2. — Data Sharing Statement [file jamanetwopen-e259143-s002.pdf]

Data Sharing Statement

Columbo. Effectiveness of Transcarotid vs Transfemoral Carotid Stenting for Stroke Prevention. *JAMA Netw Open*. Published April 25, 2025.
doi:10.1001/jamanetworkopen.2025.9143

Data
Data available: No

Additional Information
Explanation for why data not available: Our data use agreement with Medicare does not allow us to share the data used for this study. Research identifiable files are available from Medicare after execution of a data use agreement.
